# Taxonomic traits in the microstructure of flowers of parasitic *Orobanche picridis* with particular emphasis on secretory structures

**DOI:** 10.1007/s00709-019-01438-3

**Published:** 2019-09-16

**Authors:** Agata Konarska, Piotr Chmielewski

**Affiliations:** 1 Department of Botany and Plant Physiology, Faculty of Horticulture and Landscape Architecture, University of Life Sciences in Lublin, Akademicka 15, 20-950 Lublin, Poland; 2 Zamość Wildlife Association, Partyzantów 74/59, 22-400 Zamość, Poland

**Keywords:** Anatomy and ultrastructure, Histochemistry and fluorescence, Parasitic broomrape, Secondary metabolites, Secretory structures

## Abstract

*Orobanche picridis* is an obligate root parasite devoid of chlorophyll in aboveground organs, which infects various *Picris* species. Given the high level of phenotypic variability of the species, the considerable limitation of the number of taxonomically relevant traits (mainly in terms of generative elements), and the low morphological variation between species, *Orobanche* is regarded as one of the taxonomically most problematic genera. This study aimed to analyse the taxonomic traits of *O. picridis* flowers with the use of stereoscopic and bright-field microscopy as well as fluorescence, scanning, and transmission electron microscopy. The micromorphology of sepals, petals, stamens, and pistils was described. For the first time, the anatomy of parasitic *Orobanche* nectaries and the ultrastructure of nectaries and glandular trichomes were presented. Special attention was paid to the distribution and types of glandular and non-glandular trichomes as well as the types of metabolites contained in these structures. It was demonstrated that the nectary gland was located at the base of the gynoecium and nectar was secreted through modified nectarostomata. The secretory parenchyma cells contained nuclei, large amyloplasts with starch granules, mitochondria, and high content of endoplasmic reticulum profiles. Nectar was transported via symplastic and apoplastic routes. The results of histochemical assays and fluorescence tests revealed the presence of four groups of metabolites, i.e. polyphenols (tannins, flavonoids), lipids (acidic and neutral lipids, essential oil, sesquiterpenes, steroids), polysaccharides (acidic and neutral polysaccharides), and alkaloids, in the trichomes located on perianth elements and stamens.

## Introduction

Parasitic angiosperms occur in tropical forests and arctic islands. They comprise over 4000 dicotyledonous species representing 22 families (Nickrent 2002). One of them is the family Orobanchaceae, which includes, with few exceptions, about 90 genera of facultative and obligate hemiparasities and holoparasites, as specified by the APG system (2009, 2016). The genus *Orobanche* L. is the most numerous taxon in the family Orobanchaceae with approx. 200 species of obligate root parasites devoid of chlorophyll in aboveground organs (Uhlich et al. 1995; Pusch and Günther 2009). Many of the taxa are parasites of crop plants from the families Apiaceae, Asteraceae, Brassicaceae, Cucurbitaceae, Fabaceae, and Solanaceae growing in Eastern Europe, the Mediterranean region, Western Asia, as well as Cuba and California, where *Orobanche* spp. infestation has been detected in an area of several hundred thousand hectares (Sauerborn 1991; Riches and Parker 1995; Parker 2009). Some *Orobanche* species infect a single host species or genus, while others parasitise many species and genera (Thieret 1971). There are also species that parasitise the same host plant (Kreutz 1995).

In Poland, 18 species of broomrapes (Zając and Zając 2001; Halamski 2005; Piwowarczyk 2011) have been described to date, with a majority classified as rare species, as in other European countries (e.g. the Czech Republik, Germany, Romania, Slovakia), and are hence threatened with extinction (Korneck et al. 1996; Feráková et al. 2001; Procházka 2001; Höniges et al. 2008; Zarzycki et al. 2014).

Pink, yellowish, orange, beige, reddish-violet to purple, or blue *Orobanche* inflorescence shoots emerge from the ground from spring to autumn and grow quickly at a rate up to several centimetres per day (Marudagan 1950). The shoots and flowers of many species bear secretory trichomes characterised by varied density (Kreutz 1995). The zygomorphic, two-lipped, tubular flowers of broomrapes are adapted to insect pollination, although they are capable of partial self-pollination as well (Kuijt 1969; Thieret 1971; Kreutz 1995). Bumblebees, Colletid and Halictid bees, wasps, and stiletto flies visit *Orobanche* flowers from early morning to late afternoon (Jones 1991; Toth et al. 2013). The flowers attract insects with their fragrance (Hegnauer 1990; Piwowarczyk et al. 2015; Tóth et al. 2016) and offer pollen and nectar reward (Saghir and Dastgheib 1978; Ollerton et al. 2007). Annular nectaries in *Orobanche* flowers are located at the base of the ovary (Kreutz 1995; Bekker and Kwak 2005; Fahmy 2013). The nectar of the parasite usually exhibits a lower concentration than that in the host plant (Ollerton et al. 2007).

Many *Orobanche* species are characterised by slight morphological variations; hence, they are difficult to distinguish in terms of taxonomy. The presence of the host plant may facilitate to some extent identification of the *Orobanche* species growing nearby; however, the host plant sometimes grows at a distance of even several meters away from the parasite (Kreutz 1995). Furthermore, due to the high phenotypic variability, the significant reduction of the number of potential taxonomically relevant traits (mainly generative features), and the loss of colour during desiccation of herbarium specimens, *Orobanche* is one of the most difficult genera for taxonomic classification (Foley 1998, 2001; Schneeweiss et al. 2009).

*Orobanche picridis* F.W. Schultz is a characteristic species of the flora of Europe, also growing in Asia and Africa, which infects different *Picris* species, in particular *Picris hieracioides* (Piwowarczyk 2012). As shown by the Polish Red Book of Plants, this is a rare species threatened with extinction (Zarzycki et al. 2014). In the Polish conditions, it flowers in June and July and prefers initial habitats located on heavy carbonate rendzinas, e.g. fallows, mid-field fallows, field margins, abandoned fields, orchards, and pastures. The species is also common in xerothermic grasslands and scrubs and on sunny slopes (Zając and Zając 2001; Piwowarczyk 2011, 2012).

Previous studies on the taxonomic traits in different *Orobanche* spp. were conducted by Sbaih Abu et al. (1994), Plaza et al. (2004), Piwowarczyk et al. (2014, 2015), Zare et al. (2014), and Piwowarczyk and Kasińska (2017), who analysed the micromorphology of corolla petals, pollen grains, and seeds. The aim of the present investigations was to identify other taxonomically important traits in the microstructure of *O. picridis* flowers that can be useful for the diagnostics of the genus *Orobanche*. Special attention was devoted to the location and structure of floral nectaries and glandular trichomes, i.e. traits that are regarded to be helpful in studies of the taxonomy (relatedness between taxa) and evolution of plants (Behnke 1984; Galetto and Bernardello 2004; Schilmiller et al. 2008; Hassan and El-Awadi 2009; Antoń and Kamińska 2015; Konarska 2015, 2017), and have great importance for the broad-sense ecology of the species, especially the floral biology and interactions between the plant and the pollinator (Anderson 1995; López and Galetto 2002; Silva et al. 2016). The ultrastructural studies of *O. picridis* nectaries present for the first time the function (mode of nectar production and transport) of these glands in the group of parasitic plants. Additionally, the glandular trichomes were analysed for their content of secondary metabolites, whose presence may also have taxonomic relevance (Adedeji et al. 2007; Muravnik et al. 2019).

## Material and methods

*Orobanche picridis* (F.W. Schultz) inflorescence shoots were observed in June 2013 and 2018 in initial xerothermic grasslands in the “Machnowska Góra” reserve, Poland (50°22′23″N, 23°34′51″E).

During the full flowering and nectar release phase, five newly opened flowers were collected from each of 10 selected inflorescence shoots. To ensure that they were at the same stage of development, the flowers were collected successively (2–3 times) along the opening process. Preliminary morphological observations of the flowers were carried out using an SMT 800 stereoscopic microscope coupled with a NIKON COOLPIX 4500 camera. Hand-made microscopic slides were made from fresh plant material and viewed in water (calyx, corolla, stamens, and pistils) and after application of various histochemical assays and fluorochromes (calyx, corolla, and stamens) using a Nikon SE 102 light microscope and a Nikon 90i fluorescence microscope equipped with a digital camera (Nikon Fi1) and NIS-Elements Br 2 software, respectively. The distribution and structure of the glandular and non-glandular trichomes located on perianth elements and stamens and the presence of biologically active compounds in these structures were analysed.

Herbarium specimens supporting this study have been placed in the Herbarium of the Maria Curie-Sklodowska University in Lublin (LBL P) under number 1000.

### Scanning electron microscopy

For SEM analyses, fragments of sepals, corolla petals, and pistils were fixed in 4% glutaraldehyde in 0.1 M phosphate buffer with a pH of 7.0. The samples were dehydrated in an ethanol series and dried at the critical point in liquid CO_2_ (Bal-Tec CPD 030 critical point dryer). Afterwards, they were coated with gold-palladium using an EMITECH K 550x sputter coater. The preparations were observed under a TESCAN/VEGA LMU scanning electron microscope at an accelerating voltage of 30 kV. The number of stomata per square millimetre (*n* = 10) in the nectary epidermis and the length of glandular and non-glandular trichomes (*n* = 20) were counted with the use of morphology software coupled with SEM.

### Light microscopy

To obtain semi-thin sections, fragments of ovaries with nectaries and sepals with trichomes (4 × 4 mm, *n* = 5) were fixed in 2.5% glutaraldehyde in 0.1 M phosphate buffer at pH 7.2 for 12 h at a temperature of 4 °C. Next, they were carefully washed three times in phosphate buffer, dehydrated in an ethanol series, and embedded in LR white resin (LR white acrylic resin, medium grade, Sigma-Aldrich). Semi-thin sections, with a thickness in the range of 70–80 μm, were cut with glass knives using a Reichert Ultracut S ultramicrotome. For general histology, the semi-thin sections were stained with a 1% aqueous methylene blue-azure II solution (O’Brien and McCully 1981). The presence of water-insoluble polysaccharides was detected using Periodic acid-Schiff’s (PAS) reagent (O’Brien and McCully 1981) after blocking of free aldehyde groups.

### Histochemistry and fluorescence

The following histochemical assays were used: ferric trichloride (Johansen 1940; Gahan 1984) and Toluidine Blue O (Gutmann 1995) for polyphenols, potassium dichromate (Gabe 1968) to detect tannins, Sudan IV and Sudan Red B (Pearse 1985; Brundrett et al. 1991) for stain total lipids, Nile Blue (Cain 1947; Jensen 1962) for neutral and acidic lipids (essential oil), Nadi reagent (David and Carde 1964) for terpenes (essential oil), concentrated sulphuric acid (Geissmann and Griffin 1971; Cappelletti et al. 1986) for sesquiterpenes, Ruthenium Red (Johansen 1940; Jensen 1962) for presence of acidic polysaccharides, PAS reagent (O’Brien and McCully 1981) for neutral polysaccharides, and Wagner reagent (Furr and Mahlberg 1981) for alkaloids.

Fluorescence microscopy was used for determination of the location of lipids, essential oil, flavonoids, steroids, and polyphenols after application of various fluorochromes; Neutral Red for essential oil (Kirk 1970; Conn 1977); aluminium chloride and magnesium acetate (Charrière-Ladreix 1976) for flavonoids; antimony trichloride (Jensen 1962; Buttkus et al. 1977) for terpenes contain steroids; and UV light (autofluorescence) for essential oil. Fluorescence was observed using a Cy5 filter set (excitation light of 590–650 nm and a barrier filter wavelength 663–738 nm), a FITC filter set (excitation light of 465–495 nm and a barrier filter wavelength 515–555 nm), and a TRITC filter set (excitation light of 525–565 nm and barrier filter wavelength 555–600 nm). The histochemical methods were used in compliance with standard control procedures suggested by the various authors.

### Transmission electron microscopy

Fragments of ovaries with nectaries and sepals with trichomes (2 × 2 mm, *n* = 5) fixed as described above were treated for 1.5 h with 1% osmium tetraoxide solution at 0 °C and washed three times in distilled water. Next, samples were dehydrated with a graded ethanol series and embedded in LR white resin (as in LM methods). Samples were cut in the Reichert Ultracut S microtome into ultra-thin sections of 80 nm, subsequently stained with 0.5% uranyl acetate and post-stained in 0.5% lead citrate (Reynolds 1963). Then, the sections were examined with a JEM 1400 (JEOL Co., Japan) transmission electron microscope at an accelerating voltage of 120 kV equipped with 11 Megapixel TEM Camera MORADA G2 (EMSIS GmbH, Germany).

## Results

### Flower micromorphology

**Fig. 1:**
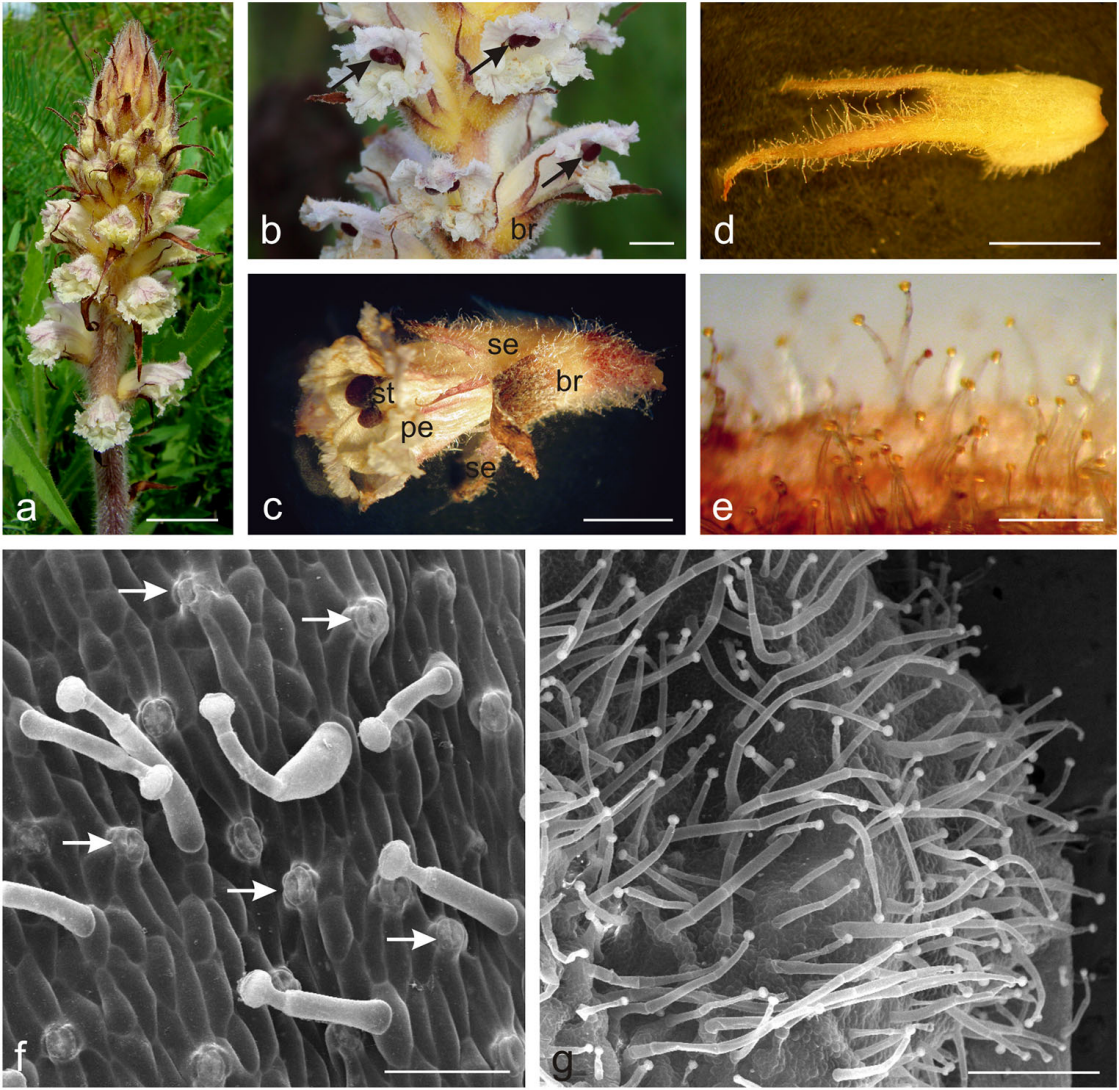
Micromorphology of *O. picridis* flowers. **a** Inflorescence. **b** Flowers in the anthesis period. Note red stigmas (*arrows*). **c** Flowers with glandular trichomes visible on bracts, sepals, and petals. **d** Sepal with numerous glandular trichomes on its abaxial side. **e** Fragment of the sepal with glandular trichomes with yellow heads. **f** Abaxial surface of the sepal with glandular trichomes and stomata (*arrows*). **g** Numerous glandular trichomes on the abaxial surface of the petal; *st* stigma, *br* bract, *se* sepals, *pe* petals. Scale bars = 2 cm (**a**), 5 mm (**b**–**d**), 500 μm (**e**, **g**), 100 μm (**f**)

The zygomorphic *Orobanche picridis* flowers developed on the inflorescence shoots in the axilla of brown-yellow bracts (Fig. 1a–c). Two ca. 1.7-cm long free sepals were divided into two lateral segments up to half their length (bipartite). The lower part of the sepal was ovoid and light yellow, whereas the upper part was dark brown and terminated with two to three filiform teeth (Fig. 1d). The ca. 2-cm long creamy-white coloured corolla with purple venation and an open throat had a lower lip with three curved, strongly ragged lobes with an undulating margin and an arcuate upper lip with two lobes with a serrated margin (Fig. 1a–c). The corolla tube was slightly longer than the sepals and slightly narrowed at the stamen attachment site. The abaxial surface of the sepals and, to a lesser extent, the corolla petals were covered with yellow-headed glandular trichomes (Fig. 1d–g). Numerous stomata were visible in the epidermis of the abaxial surface of the sepals, whose cells were covered with a smooth cuticle (Fig. 1f). The glandular trichomes had varied length: from 0.78 to 1.68 mm on the sepals and from 0.78 to 0.98 mm on the corolla petals (Fig. 1c–g). There were a few similar glandular trichomes on the adaxial surface of the upper lip at the height of the stigma.

**Fig. 2:**
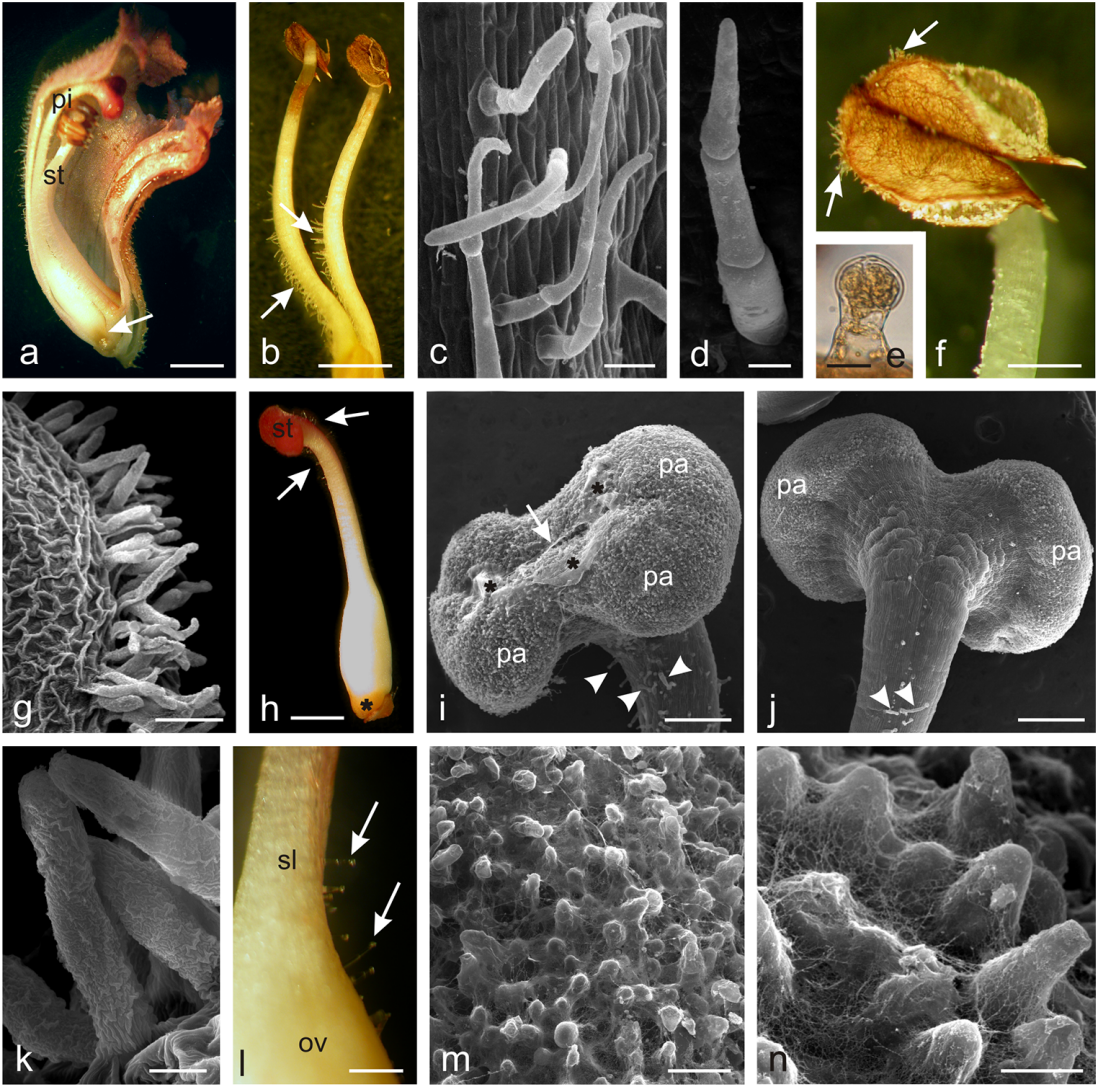
Micromorphology of *O. picridis* stamens and pistils. **a** Longitudinal section of the flower with visible stamens (*st*), pistil (*pi*), and nectary (*arrow*). **b** Stamens with visible non-glandular trichomes (*arrows*) on their filaments. **c** Non-glandular trichomes on the stamen filaments. **d** Three-cellular non-glandular trichome. **e** Glandular trichome on the stamen filament near the anther. **f** Stamen filament with visible non-glandular trichomes (*arrows*) on anthers. **g**, **k** Non-glandular trichomes located along anther sutures. **k** Note the massive cuticular striae on the trichome surface. **h** Pistil with visible nectary (*asterisk*) and glandular trichomes (*arrows*) near stigma (*st*). **i**, **j** Stigma covered with papillae (*pa*) with a visible slit (*arrows*) and viscous substance (*asterisks*) on the surface. Note glandular trichomes (*arrowheads*) on the style surface. **i** Front view. **j** Back view. **l** Fragment of the pistil style (*sl*) and ovary (*ov*) with non-glandular trichomes (*arrows*). **m**, **n** Papillae on the stigma surface covered with numerous threads of viscous substance. Scale bars = 2 mm (**a**, **b**, **h**), 500 μm (**f**, **l**), 250 μm (**i**, **j**), 100 μm (**c**, **g**, **m**), 30 μm (**d**), 20 μm (**e**, **k**), 10 μm (**n**)

The lower part of the corolla tube, i.e. at a distance of 2–3 mm from its base, had four different-length stamens, two of which were located closer to the pistil (Fig. 2a). One- to five-celled viable non-glandular trichomes with a length ranging from 0.13 to 0.44 mm were visible at 2/3 of the filament length on the side adjacent to the ovary and style (Fig. 2b–d). The trichomes were covered with a smooth cuticle and had a rounded or pointed apical part. Their greatest number and length were noted in the basal part of the filaments. The stamen filaments always had only two short (ca. 70–80 μm) glandular trichomes close to the anthers (Fig. 2e). Non-glandular trichomes with an average length of 0.14 mm covered with a striated cuticle were also found along the sutures of the beige-brown anthers (Fig. 2f–k).

The *O. picridis* flowers had one 2-carpelled pistil with an elongated ovoid ovary and a claret-stained style with a large claret stigma bent towards the lower lip (Fig. 2h). The upper part of the ovary and style had glandular trichomes with an average length of 0.15 mm (from 0.11 to 0. 37 mm); they were composed as those found on the sepals and petals (Fig. 2h–j, l). The greatest number of short glandular trichomes was located immediately under the stigma. The centrally narrowed two-lobed bow-shaped stigma in the middle part was characterised by the presence of a distinct longitudinal slit running along its longer axis (Fig. 2i). The surface of the stigma was formed of papillae covered with a viscous cobweb-like substance at the receptive stage (Fig. 2i–n). The location of non-glandular and glandular trichomes in the generative organs of *O. picridis* is presented in Table 1.

**Table 1:** Localization of non-glandular (ng) and glandular (g) trichomes in generative organs of different *Orobanche* species

Species	Stamen				Pistil				Source
	Filament		Anther		Ovary		Style		
	ng	g	ng	g	ng	g	ng	g	
*O. amethystea*	+	–	–	–	nd	nd	nd	nd	Joel and Eisenberg 2002
*O. pubescens*	+	–	–	–	nd	nd	nd	nd	
*O. loricata*	–	–	–	–	nd	nd	nd	nd	
*O. owerini*	–	+	+	–	–	+	–	–	Zare and Dönmez 2013
*O. reticulata*	–	–	–	–	–	–	–	+	
*O. iammonensis*	+	–	+	–	–	+	–	+	Pujadas-Salvà and Arguimbau 2008
*O. krylowii*	+	+	+	–	+	–	–	+	Frajman et al. 2013
*O. aegyptica*	+	+	+	–	–	–	–	+	Hassan and El-Awadi 2009
*O. crenata*	–	+	–	–	–	–	–	+	
*O. picridis*	+	+	+	–	–	+	–	+	This study

*+* present, *−* absent, *nd* not done

### Microstructure of *O. picridis* nectary

**Fig. 3:**
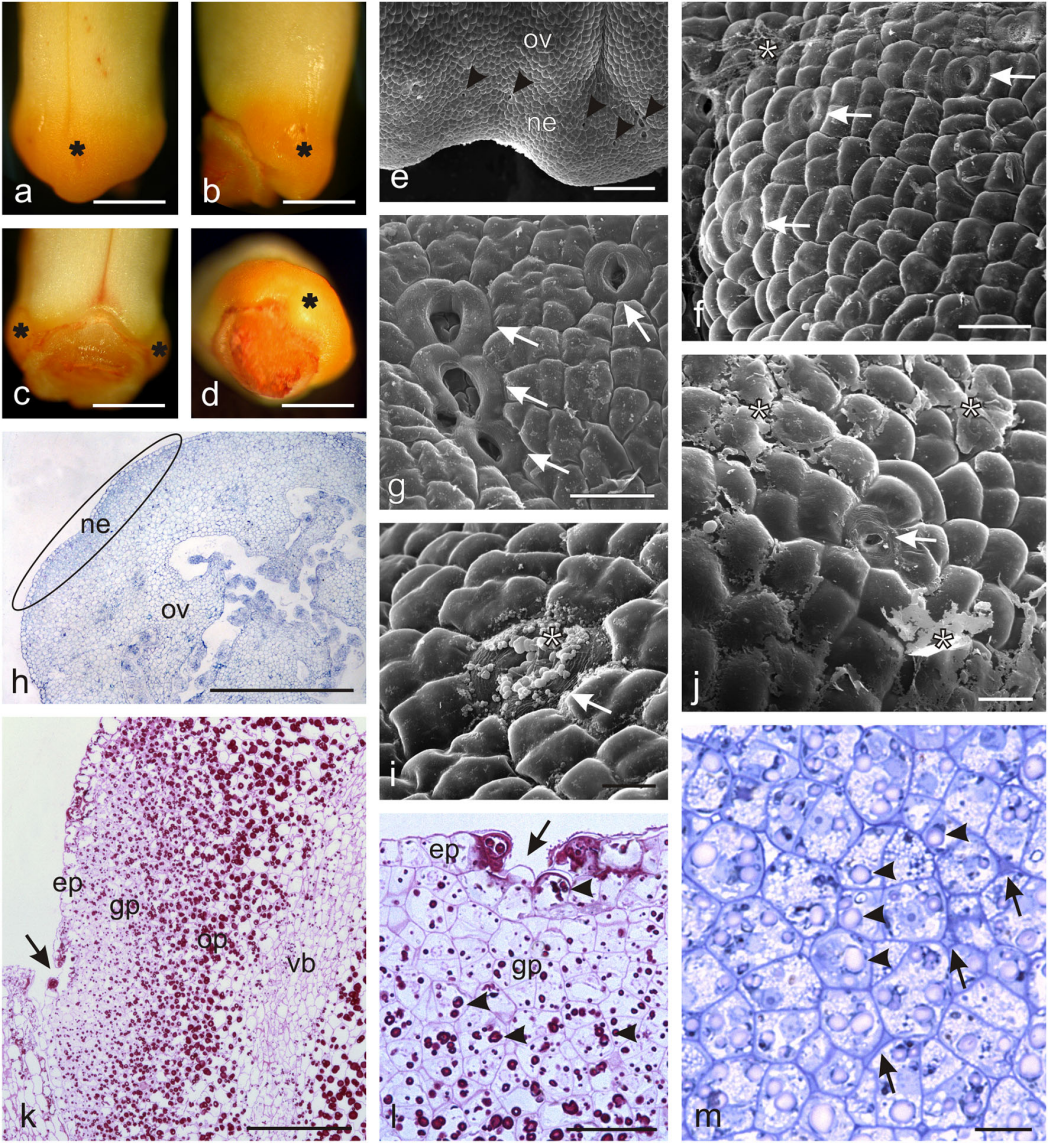
Microstructure of *O. picridis* nectaries. **a–d** Gynoecial nectary (*asterisks*) in *O. picridis* flowers. **a** View of the side of the lower lip. **b** Lateral view. **c** View of the side of the upper lip. **d** Top view. **e** Fragment of the ovary and nectary surface with nectarostomata (*arrowheads*). **f**, **g**, **i**, **j** Fragments of the nectary surface with nectarostomata (*arrows*). Note the dried secretion (*asterisks*) on the nectary surface. **h** Cross-section of an ovary with a nectary (*elipsa*). **k** Fragment of the longitudinal section of an ovary with a nectary (PAS). Note numerous starch grains in the ovary and glandular parenchyma cells. Vascular bundle visible in the ovary wall (*vb*). Nectarostoma visible in the nectary epidermis (*arrow*). **l**, **m** Fragments of longitudinal sections of nectariferous tissue. **l** Visible epidermis with nectarostoma (*arrow*) and glandular parenchyma with starch grains (*arrowheads*) (PAS). **m** Different-shaped glandular parenchyma cells with visible starch grains (*arrowheads*) and intracuticular spaces with dark content (*arrows*); *ov* ovary, *ne* nectary, *ep* epidermis, *gp* glandular parenchyma, *op* ovary parenchyma. Scale bars = 1 mm (**a**–**d**, **h**), 200 μm (**e**, **k**), 50 μm (**f**, **g**, **j**, **l**), 20 μm (**i**, **m**)

The yellow-orange *O. picridis* nectary embedded in the base of the gynoecium formed a convexity on the ovary surface (Fig. 3a–d). The gland was characterised by varied height and thickness; it was the highest and most protruding at the side of the lower lip, while its height and thickness were reduced to zero at the side of the upper lip (Fig. 3a–d). The tetragonal or pentagonal nectary epidermis cells had a convex outer wall and a smooth cuticle (Fig. 3f, g, i, j). They had a smaller outline than the adjacent ovary epidermis cells (Fig. 3e). Nectar was released through modified nectarostomata, which often formed clusters of two or three and were typically surrounded by six to eight guard cells (Fig. 3f, g, i, j). The nectarostomata were located at the level of epidermal cells or in small depressions. There were on average 64 stomata per 1 mm^2^ (± 18) of the nectary surface. A dried secretion in the form of granules or a continuous exfoliating layer was sometimes observed on the surface of the stomata (Fig. 3i, j). Secreted nectar accumulated between the ovary and the corolla tube.

The transverse and longitudinal sections of the nectary demonstrated that the gland was composed of a single-layered epidermis and several dozen layers of glandular parenchyma, whose cells exhibited compact arrangement, varied shapes, and smaller outline sizes than the adjacent ovary cells (Fig. 3h, k–m). The nectary epidermis cells were characterised by a high degree of vacuolisation, whereas the glandular parenchyma cells had small vacuoles and abundant cytoplasm (Fig. 3k–m). The epidermis cells and, to a greater extent, the glandular tissue cells contained starch grains, which stained intensively when treated with Schiff’s reagent (Fig. 3k, l). Greater numbers of larger starch grains were also observed in the ovary wall cells (Fig. 3k). The intercellular spaces in the glandular parenchyma were often filled with dark-stained content (Fig. 3m). The nectary gland did not have a vascular tissue. In turn, there were vascular bundles of the ovary walls close to the glandular tissue (Fig. 3k), which were not branched and thus unable to reach the nectary tissues.

**Fig. 4:**
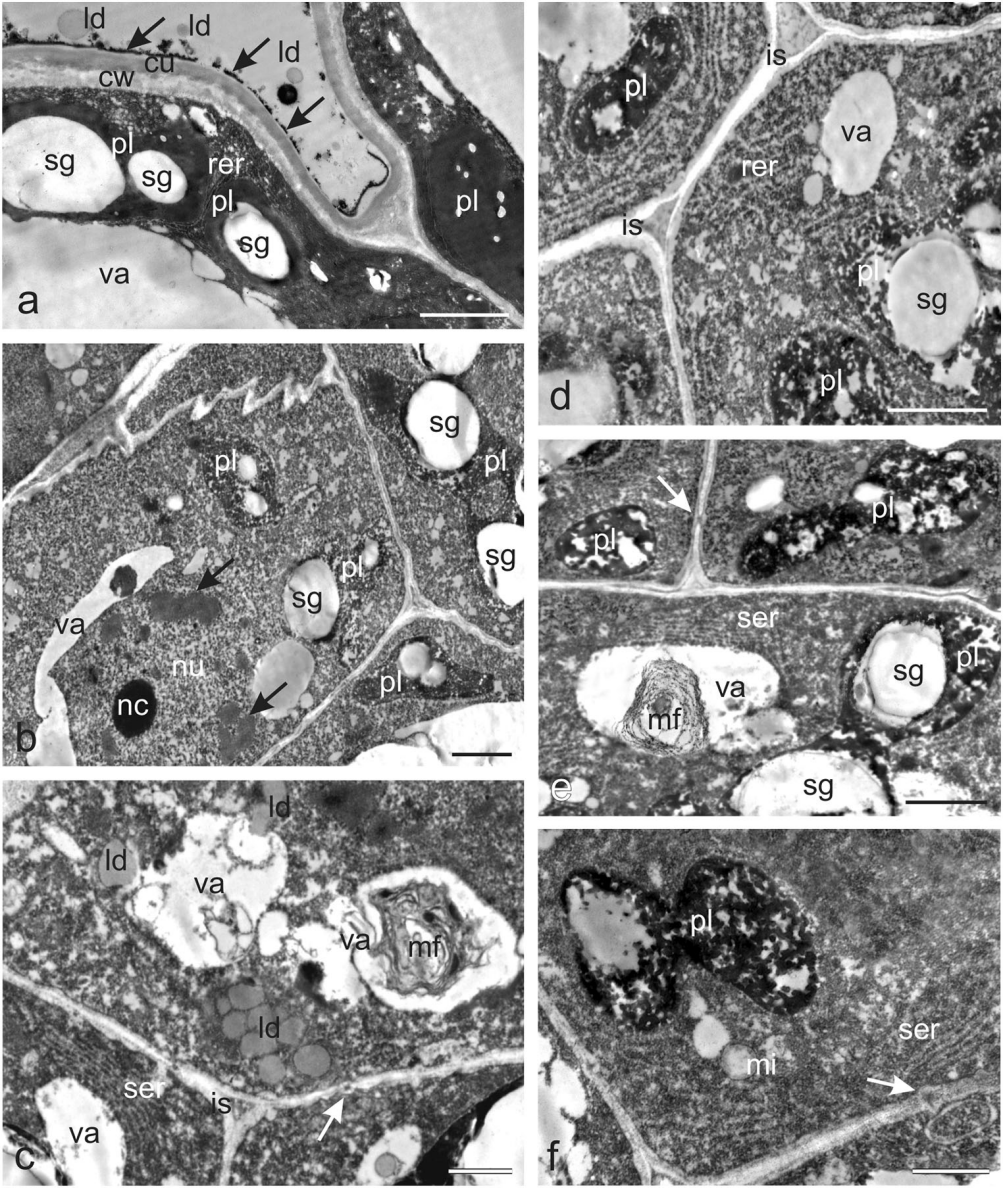
Ultrastructural traits of the *O. picridis* nectary. **a** Fragment of secretory epidermis cells. Visible dense cytoplasm with plastids filled with starch grains and dense osmiophilic material content, rer, lipid droplets, and large vacuoles. Note the thin osmiophilic film (*arrows*) on the cuticle surface (*cu*). **b–f** Cells of glandular parenchyma with dense cytoplasm, polymorphic plastids, small vacuoles, and thin cell walls. **b** Note the large nucleus with a conspicuous nucleolus and dark areas with heterochromatin (*arrows*) and plastids with starch grains and areas with transparent and osmophilic content. **c** Visible numerous lipid droplets, well-developed ser, vacuoles with myelin-like multilamellar figures, plasmodesmata (*arrow*), and intercellular space with dense content. **d** Note numerous rough endoplasmic reticulum profiles forming closely packed strands, plastids with starch grains, and intercellular space with dense content. **e** Visible plastids with large starch grains, smooth endoplasmic reticulum profiles, plasmodesmata (*arrow*) in the cell wall, and myelin-like multilamellar figures in the vacuole. **f** Note the dividing plastid, ser profiles, mitochondria, and plasmodesmata (*arrow*); *pl* plastids, *sg* starch grains, *va* vacuoles, *ld* lipid droplets, *ser* smooth endoplasmic reticulum, *rer* rough endoplasmic reticulum, *mi* mitochondria, *mf* myelin-like multilamellar figures, *is* intercellular spaces. Scale bars = 2 μm (**a**, **b**, **d**–**f**), 1 μm (**c**)

The TEM micrographs of the cytoplasm of the nectary epidermis cells demonstrated numerous endoplasmic reticulum (ER) profiles, mitochondria, and lipid droplets in addition to the starch grains in the polymorphic plastids (Fig. 4a). The plastids often exhibited dense osmiophilic material. Small lipid droplets were also observed on the nectary epidermis surface. The epidermal cells had large vacuoles, and the outer cell wall was covered with a relatively thick cuticle layer, whose surface was covered with a thin osmophilic film (Fig. 4a). The secretory parenchyma cells exhibited dense cytoplasm containing numerous organelles and the presence of primary pit fields with plasmodesmata in the cell walls (Fig. 4b–f). Plasmodesmata were also present in the walls between secretory parenchyma cells and ovary wall cells (not shown). The secretory parenchyma cells contained relatively large nuclei with conspicuous nucleoli with areas exhibiting a substantial amount of heterochromatin (Fig. 4b) and numerous different-shaped large plastids (with a size often exceeding that of the cell nucleus) containing large starch granules and characterised by the absence of thylakoids and the presence of transparent and osmophilic content (Fig. 4b, d–f). Well-developed smooth and rough ER profiles in the parietal cytoplasm were usually arranged parallel to the secretory tissue walls, thus forming closely packed strands (Fig. 4c–f). The cells contained mitochondria (Fig. 4f) and clusters of small lipid droplets (Fig. 4c) as well as vacuoles with myelin-like multilamellar figures (Fig. 4c, e). The small intercellular spaces between the secretory parenchyma cells were often filled with dark content (Fig. 4c, d).

### Microstructure of glandular and non-glandular trichomes

**Fig. 5:**
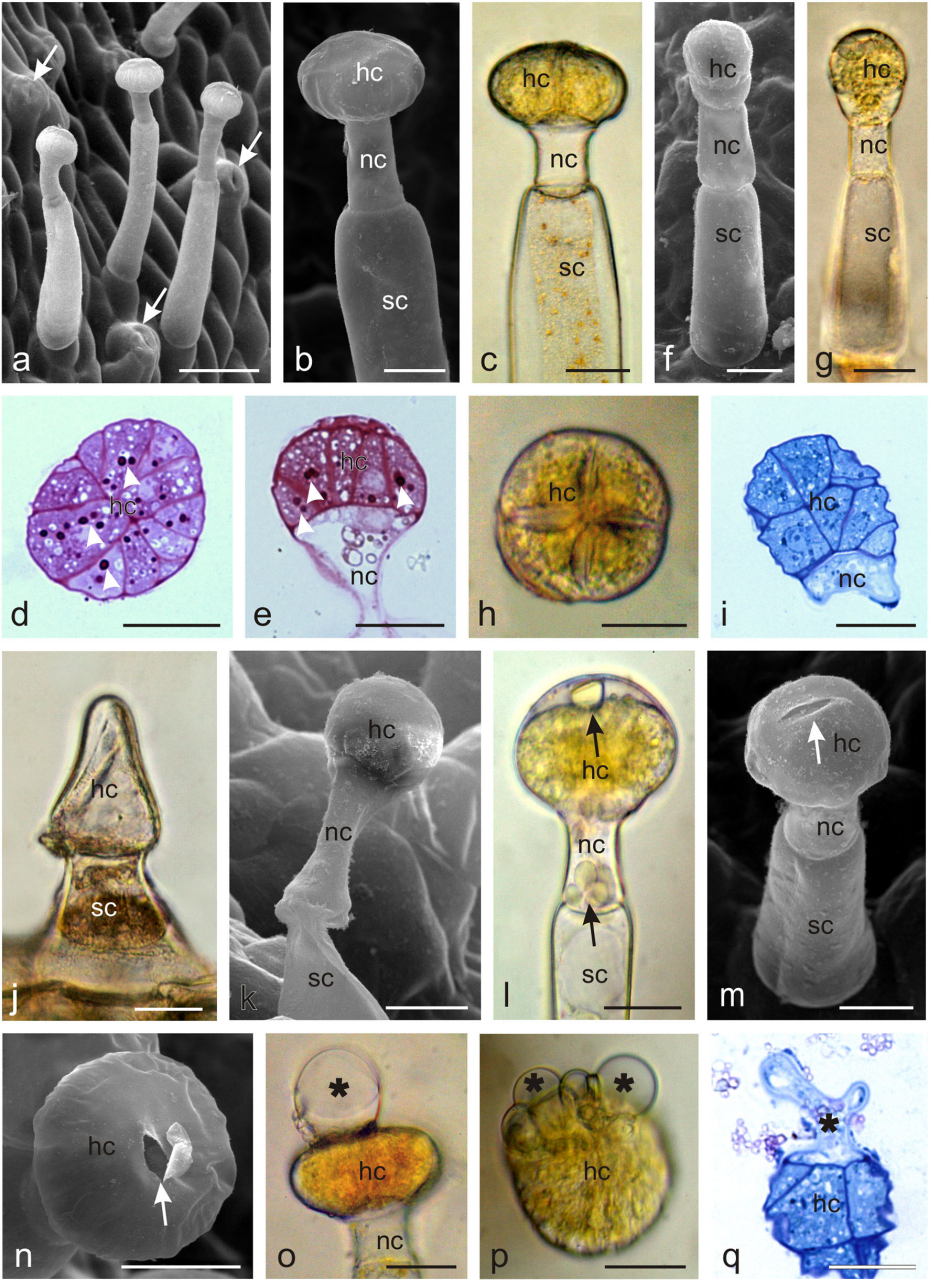
Microstructure of glandular trichomes from the abaxial surface of an *O. picridis* sepal and petal. **a** Visible capitate glandular trichomes and stomata (*arrows*). **b–e** Glandular trichomes type I with a multicellular secretory head formed by cells arranged in a circle. **d**, **e** Cross (**d**) and longitudinal (**e**) sections of glandular trichomes type I. Note the dense cytoplasm with stained starch grains (*arrowheads*) (PAS reaction) and fine vacuoles. **f–i** Glandular trichomes type II with a two-layered head. **h** Trichome type II head on the upper side. **i** Longitudinal section of trichome head type II. Visible dense cytoplasm with a large nucleus and small vacuoles. **j** Conoidal trichomes with the characteristic long conical glandular cell and a short bicellular stalk. **k** Ageing trichome type I with a narrowed neck and stalk cells. **l** Droplets of secretion (*arrows*) visible in the subcuticular space and in the neck cell. **m**, **n** Note the ruptures (*arrows*) in the cuticle on the trichome apex. **o–q** Visible trichome heads with secretion (*asterisks*) exuded from ruptures in the cuticle. **o**, **q** lateral view. **p** Top view.; *hc* head cells, *nc* neck cells, *sc* stalk cells. Scale bars = 100 μm (**a**), 50 μm (**g**, **j**), 30 μm (**b**–**f**, **h**, **i**, **k**–**q**)

Two types of capitate glandular trichomes were distinguished on the sepals and corolla petals (Fig. 5). Both trichome types were composed of a single-celled base, a several-celled (1–4) stalk, one neck cell, and a multicellular secretory head. In the first type, the head was flattened and composed of eight to several dozen secretory cells arranged in a circle, similar to orange segments (Fig. 5a–e). The other type of trichomes exhibited a more elongated head typically composed of two secretory cell layers. The upper layer was usually built of four cells (Fig. 5f–i). The heads of young and mature capitate trichomes were straw yellow, but their colour was dependent on the age of the trichomes: the heads of older trichomes were brown. During the consecutive stages of trichome development, the neck and stalk cells narrowed in older trichomes (Fig. 5k); this was followed by degeneration of the head cells, which shrank and collapsed. Both young and older trichomes produced secretion. The secretion penetrated the external wall of secretory cells in their distal part and accumulated in the subcuticular space (Fig. 5l) formed by detachment of the cuticle from the secretory cell walls. Next, the secretion was released outside through the ruptured cuticle, forming different-sized droplets/vesicles on the trichome surface (Fig. 5m–q). Additionally, conoidal trichomes with a characteristic long conical glandular cell and a short bicellular stalk were observed on the margins of the sepals (Fig. 5j). The characteristic micromorphological traits of *Orobanche picridis* trichomes and nectaries are shown in Table 2.

**Table 2:** Characteristics of trichomes and nectaries in *O. picridis* flowers

Colour of glandular trichome heads	White-yellow
Colour of glandular trichome secretion	Light yellow
Trichomes on sepals	Glandular, capitate, dense, on the abaxial surface Glandular, conoidal, rare, on margins
Trichomes on petals	Glandular, capitate, dense, on the abaxial surface
Trichomes on stamens	Non-glandular, in the bottom part of filaments, ca. 2/3 of the length Non-glandular, on anther sutures
Trichomes on the pistil	Glandular, capitate, in the upper part of ovary and style under stigma
Location of the nectary	At the base of the ovary (gynoecial nectary)
Colour of the nectary	Yellow-orange
Type of nectar exuded	Nectarostomata
Vascularisation of nectary	None
Starch grains in nectary parenchyma	Present, numerous
Nectar transport	Via symplastic and apoplastic routes

**Fig. 6:**
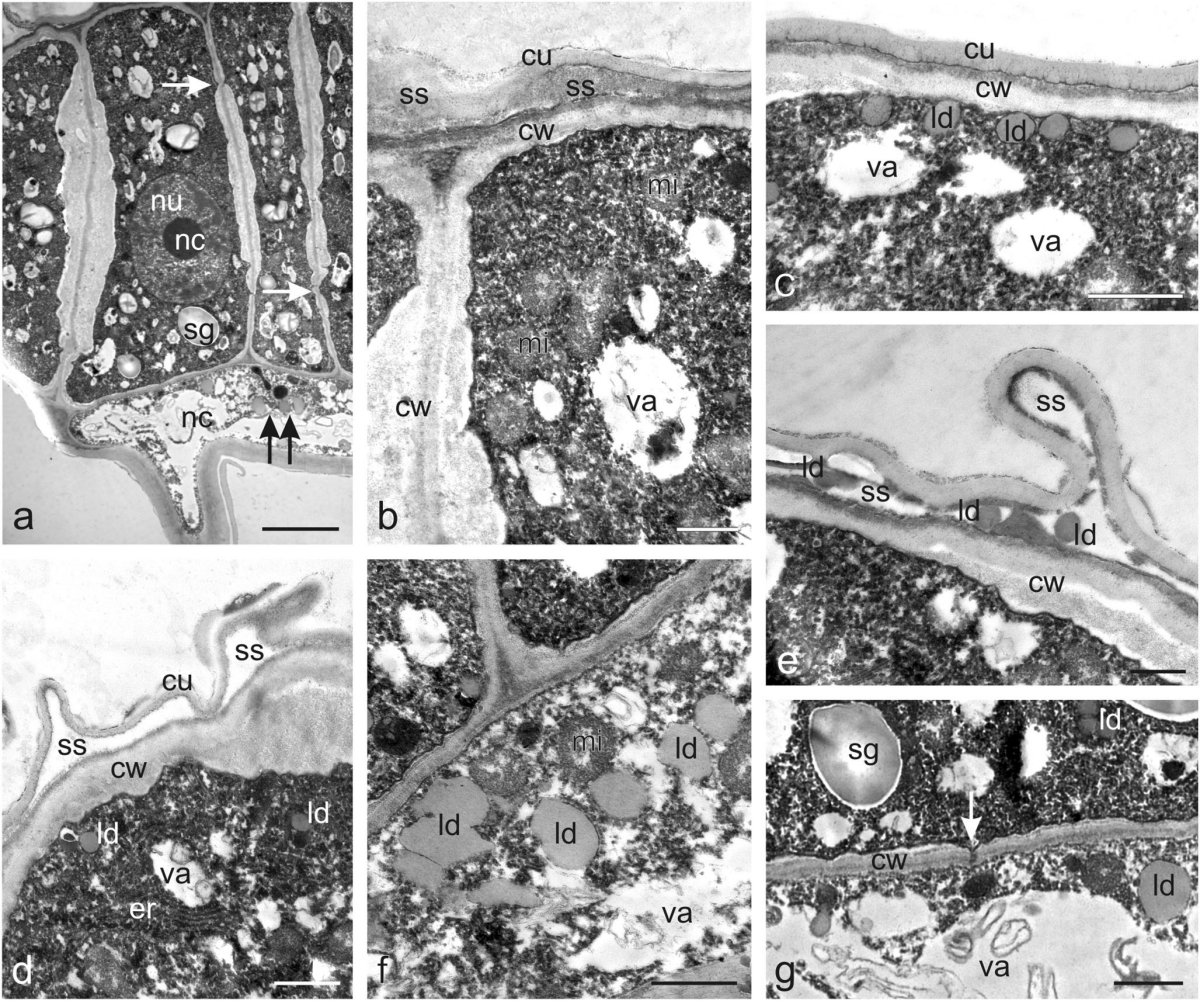
Ultrastructural traits of capitate glandular trichomes (type I) on the *O. picridis* sepal. **a** Fragment of longitudinal section of trichome head and neck cells. Note the dense cytoplasm with starch grains in the plastids, the large nucleus with a prominent nucleolus, small vacuoles, and plasmodesmata (*arrows*) in secretory cell walls. Lipid droplets visible in the neck cell (*two arrows*). **b** Apical part of the secretory cells of the trichome head with numerous mitochondria and protruding cuticle forming subcuticular space. **c** Apical fragment of a glandular head cell with numerous lipid droplets visible under the outer cell wall. **d** Apical fragment of a glandular head cell with visible endoplasmic reticulum profiles and protruding cuticle forming subcuticular space. **e** Lipid droplets visible in the subcuticular space. **f** Fragment of a neck cell with visible mitochondria and lipid droplets. **g** Visible plasmodesmata (*arrow*) connecting the head cell and the neck cell. **b**, **d**, **f**, **g** Some membranous remnants visible in vacuoles. *hc* head cells, *nc* neck cells, *sg* starch grains, *nu* nucleus, *nc* nucleolus, *va* vacuoles, *cw* cell walls, *mi* mitochondria, *cu* cuticle, *ss* subcuticular space, *ld* lipid droplets, *er* endoplasmic reticulum. Scale bars = 5 μm (**a**), 2 μm (**g**), 1 μm (**b**–**f**)

The secretory cells of the two types of trichome heads viewed in transverse and longitudinal sections during the secretory activity phase under the LM and TEM microscopes were filled with dense cytoplasm and were characterised by a low level of vacuolisation (Figs. 5d, e, i, q, 6a–f). Some vacuoles were empty and electron translucent, while others contained some membranous remnants. The treatment with PAS indicated the presence of numerous starch grains in the secretory cells of the trichome heads (Fig. 5d, e). Besides plastids with starch grains, the cytoplasm contained relatively large cell nuclei with prominent nucleoli (Fig. 6a), numerous mitochondria (Fig. 6b, f), ER profiles (Fig. 6d), and lipid droplets (Figs. 6c, e–g). The latter accumulated mainly near the plasmalemma of the peripheral head cells, in the subcuticular space at the apex of the trichomes, and in the trichome neck cells (Fig. 6c, e, f). The cell walls between the secretory cells and the space between the secretory cells and the neck cells exhibited pit fields with plasmodesmata (Fig. 6a, g).

### Histochemistry and fluorescence tests of trichomes

The results of histochemical assays and fluorescence tests demonstrated that the capitate glandular and non-glandular trichomes present on the sepals, petals, and filaments contained various groups of metabolites, i.e. polyphenols (tannins, flavonoids), lipids (acidic and neutral lipids, essential oil), polysaccharides (acidic and neutral polysaccharides), and alkaloids (Table 3).

**Table 3:** Primary and secondary metabolites identified by histochemical and fluorescence tests in the trichomes of *Orobanche picridis* flowers

Metabolites	Test	Calyx			Corolla			Stamens	Colour observed
		Capitate glandular trichomes			Capitate glandular trichomes			Non-glandular trichomes	
		Head cells	Neck cells	Stalk cells	Head cells	Neck cells	Stalk cells		
Polyphenols	FeCl_3_ Toluidine Blue O	++ ++	+ ++	+ +	++ ++	+ ++	+ +	+ –	Dark brown Blue, violet, turquoise
Tannins Total lipids	Potassium dichromate	++	+	+	++	+	+	+	Brown
	Sudan Red B and Sudan IV	+	+	+	+	+	+	+	Orange
Acidic and neutral lipids (essentials oil)	Nile Blue	+	+	+	+	+	+	+	Blue, pink
Essential oil	Nadi reagent	–	–	–	–	–	–	–	Dark blue, dark violet
Sesquiterpenes	Conc. H_2_SO_4_	+	+	–	+	+	–	–	Yellow
Lipids (essential oil)	Neutral Red under UV	+	++	–	+	++	–	–	Yellow
Terpenes contains steroids	Antimone trichloride under UV	–	+	–	–	+	–	–	Yellow
Lipids (essential oil)	UV-autofluorescence	+	+	–	+	+	–	+	Yellow
Acidic polysaccharides	Ruthenium Red	+	–	–	+	–	–	–	Red
Neutral polysaccharides	Periodic acid-Schiff’s reagent	+	+	+	+	+	+	+	Pink
Alkaloids	Wagner reagent	++	+	+	++	+	+	–	Brown
Flavonoids	Aluminium chloride and magnesium acetate under UV	++	+	+	++	+	+	+	Yellow

*++* intensive, *+* positive, *−* negative

**Fig. 7:**
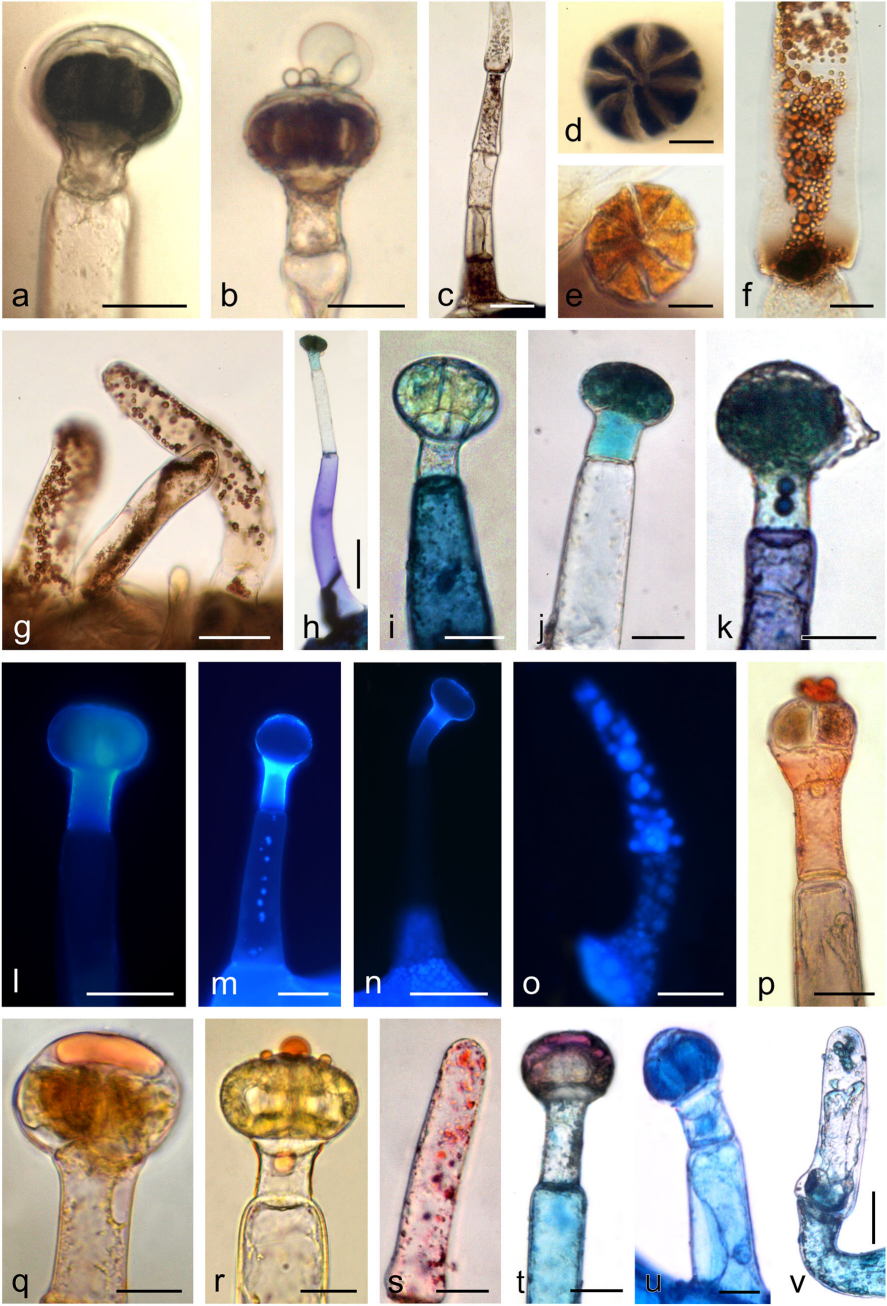
Histochemical and fluorescence tests of glandular (**a**, **b**, **d–f**, **h–n**, **p–r**, **t**, **u**) and non-glandular trichomes (**c**, **g**, **o**, **s**, **v**) in *O. picridis*. **a–d** Staining of polyphenols with FeCl_3_. **e–g** Staining of tannins with potassium dichromate. **h–k** Staining of polyphenols with Toluidine Blue O. **l–o** Fluorescence of flavonoids with aluminium chloride (**l**, **m**) and magnesium acetate (**n**, **o**) fluochromes in the Cy5 filter set. **p–s** Staining of lipids with Sudan Red B (**p**, **s**) and Sudan IV (**q**, **r**). **t–v** Staining of neutral (**t**) and acidic (**u**, **v**) lipids with Nile Blue. Scale bars = 100 μm (**n**), 50 μm (**c**, **g**, **h**, **l**, **m**, **o**), 30 μm (**a**, **b**, **i**–**k**, **p**, **s**–**u**), 20 μm (**d**–**f**, **q**, **r**, **v**)

The reaction with ferric chloride and potassium dichromate revealed the presence of phenolic compounds and tannins in the head, neck, and stalk cells of glandular trichomes located on the sepals and corolla petals (Fig. 7a–f). Polyphenols and tannins were also present in the non-glandular trichomes located on the filaments, where they formed brown different-sized droplets (Fig. 7g). Polyphenols contained in the glandular trichomes stained with Toluidine Blue O exhibited different shades of blue, navy blue, and turquoise (Fig. 7h–k). Most frequently, the trichome heads and necks were turquoise-green or turquoise, while the trichome base was purple-navy blue. In turn, droplets of a blue or navy blue liquid were sometimes visible in the stalk cells. The non-glandular trichomes on the stamens were colourless after the treatment with Toluidine Blue O. In the presence of the aluminium chloride and magnesium acetate fluorochromes, flavonoids present in the head, neck, and/or base cells of the glandular trichomes and in the non-glandular trichomes located on the stamens exhibited yellow secondary fluorescence under UV (Fig. 7l–o).

All parts of the glandular trichomes and the stamen non-glandular trichomes contained lipids, which stained positively in the Sudan Red and Sudan IV reaction (Fig. 7p–s). Different-sized orange droplets of lipophilic compounds were most often detected in the subcuticular space and in the heads of some trichomes. Similarly, the Nile Blue reaction revealed the presence of acidic lipids in the head, neck, and base cells in the glandular trichomes and the presence of neutral lipids (essential oil) present mainly in the secretion visible in the subcuticular space (Fig. 7t, u). The secretion was blue-turquoise in some trichomes and pink in others. In turn, the trichome base cells were more or less intense blue, turquoise-blue, or navy blue-pink. A positive reaction with Nile Blue was also demonstrated in the case of the stamen non-glandular trichomes (Fig. 7v).

**Fig. 8:**
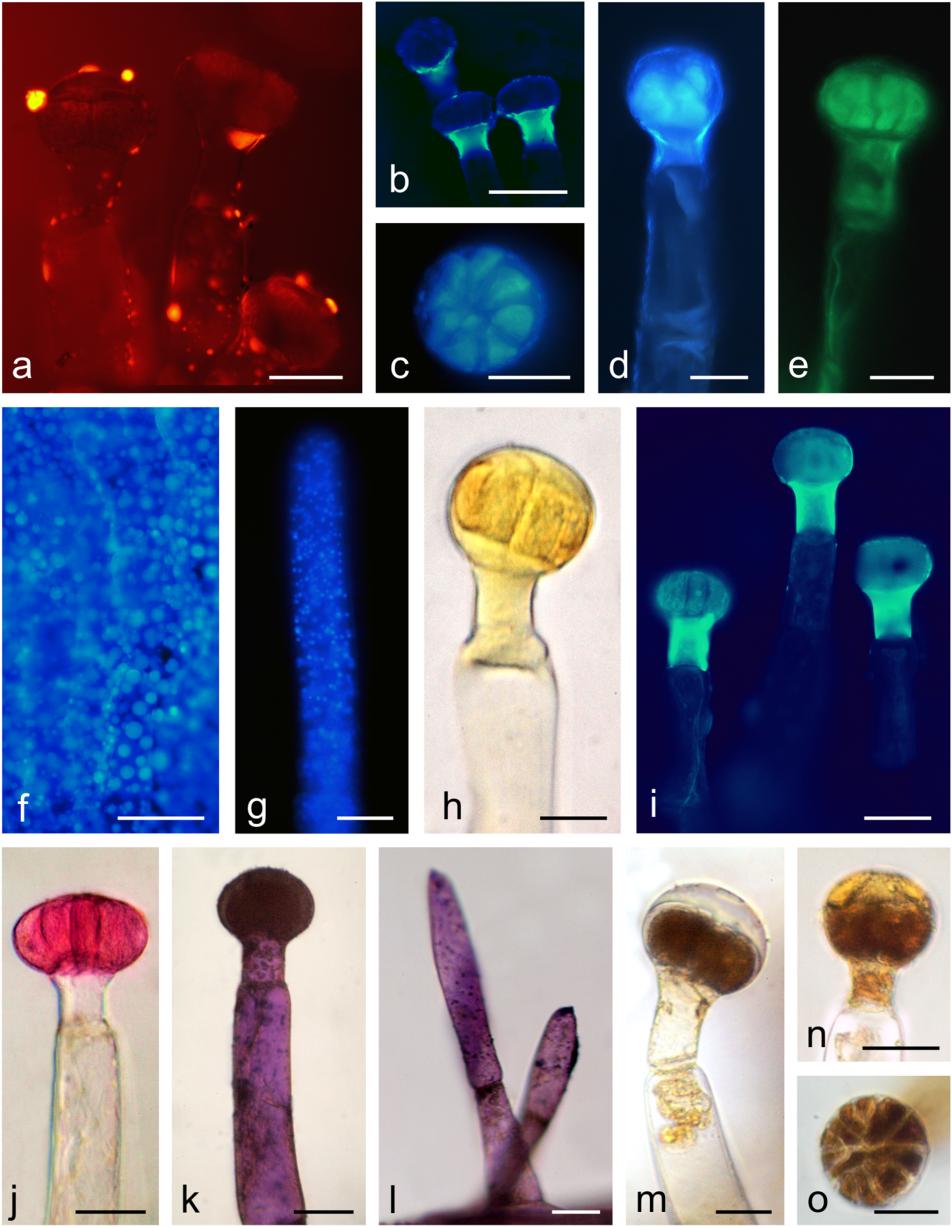
Histochemical and fluorescence tests of glandular (**a–e**, **h–l**, **m–o**) and non-glandular trichomes (**g**, **l**) in *O. picridis*. **a**, **b** Fluorescence of essential oil with Neutral Red fluochrome in the TRITC (**a**) and Cy5 (**b**) filter sets. **c–g** Autofluorescence of essential oil in the Cy5 (**c**, **d**, **f**, **g**) and TRITC (**e**) filter sets. **f** Cells of sepal epidermis. **h** Staining of sesquiterpenes with conc. H_2_SO_4_. **i** Fluorescence of terpenes contain steroids with antimony trichloride in the Cy5 filter set. **j** Staining of acidic polysaccharides with Ruthenium Red. **k**, **l** Staining of neutral polysaccharides with Periodic acid-Schiff’s reagent (PAS reaction). **m–o** Staining of alkaloids with Wagner reagent. Scale bars = 100 μm (**b**, **f**), 50 μm (**d**, **i**, **l**), 30 μm (**a**, **c**, **e**, **g**, **h**, **j**, **k**, **m**–**o**)

Under UV, Neutral Red induced secondary fluorescence of essential oil in trichome necks and droplets of secretion visible mainly on the surface of the trichomes (Fig. 8a, b). Similarly, intense autofluorescence of essential oil was observed in the heads and necks of the glandular trichomes, in non-glandular trichomes on the stamens, and in the epidermis cells of the sepals and corolla petals (Fig. 8c–g). Sesquiterpenes present in the heads and necks of the glandular trichomes stained lemon yellow after treatment with concentrated sulphuric acid (Fig. 8h). The application of the antimony trichloride fluorochrome induced secondary fluorescence of steroids mainly in the trichome necks and in the head cells (Fig. 8i). The reactions after the application of the Nadi reagent were negative.

Polysaccharides present in the head cells of the glandular trichomes stained pink when treated with Ruthenium Red, similar to the polysaccharides contained in the non-glandular trichomes present on the stamens (Fig. 8j). In turn, Schiff’s reagent yielded a cyclamen-pink colour of polysaccharides present in the head and base cells of the glandular trichomes and in the non-glandular trichomes located on the stamens (Fig. 8k, l).

In the presence of the Wagner reagent, alkaloids contained in the head, neck, and/or base cells stained dark brown (Fig. 8m–o). In some trichomes, the secretion present in the subcuticular space stained yellow as well (Fig. 8n, o).

## Discussion

### Flower and nectary microstructure

The *Orobanche picridis* flowers exhibited specific diagnostic traits in their morphology, i.e. the size and shape of corolla petals and sepals, the colour of the perianth, stigmata, and trichomes, location and colour of the nectaries, and especially, the distribution and types of non-glandular and glandular trichomes (Tables 1 and 2).

The two free sepals in *O. picridis* were slightly shorter than the corolla and were divided into two lateral halves with subulate teeth. A calyx with two lateral segments is characteristic for species from the section *Orobanche* (e.g. *O. picridis*, *O. crenata*, *O. cernua*, *O. cumana*). In turn, it is campanulate (bell shaped) in representatives of the section *Trionychon* (e.g. *O. arenaria*, *O. purpurea*, *O. ramosa*, *O. nana*, *O. caesia*) (Pujadas-Salvá 2002; Plaza et al. 2004). As demonstrated by Kreutz (1995), the length, shape, and depth of indentation of sepals are important taxonomic features facilitating identification of *Orobanche* species, similar to the size and shape of corolla. The *O. picridis* flowers had a creamy-white coloured corolla with purple venation. Many scientists report that the colour of different *Orobanche* spp. flowers may vary from white through creamy, yellow, and brown, to red, purple, blue, or violet (e.g. Pujadas-Salvá and Velasco 2000; Joel and Eisenberg 2002; Hyun et al. 2003; Mohamed and Musselman 2008). Furthermore, many researchers have reported that the two-lipped corolla with a long tube usually curved towards the lower lip is a characteristic trait of species from the section *Orobanche* (Restuccia et al. 2009; Zare and Dönmez 2013), which was observed in *O. picridis* in the present study as well.

The *O. picridis* stigmata were always dark red, had an elongated slit, and were composed of different-sized papillae. The colour and microstructure of stigmata is highly important trait in the taxonomy of the genus *Orobanche* (Kreutz 1995). However, as shown by Zare and Dönmez (2013), the colour of the pistil in *Orobanche* may differ between species or within one species. The authors observed pink or yellow pistil stigmata in *O. owerini* and yellow, brown, red, or red-violet colour of this floral part in *O. reticulata*. As suggested by Harborne (1997a), the stigma colour acts as a signal attractant associated with adaptation of plants to various types of pollinators, which see the same colours in different ways and regard different colours as attractive. Since *Orobanche* flowers are visited by many insect groups, e.g. bumblebees, Colletid and Halictid bees, wasps, and stiletto flies, the different colours of stigmas serve as attractants of different insects. Moreover, it is known that the stigma colour may change during anthesis, which is most often associated with loss of its receptivity (Armstrong and Irvine 1989; Serrano et al. 2010).

*O. picridis* has gynoecial nectaries located at the base of the elipsoid ovary forming an asymmetrical yellow gland with varied thickness and height. A similar location and colour of nectaries in other representatives of the family Orobanchaceae has been described by Bekker and Kwak (2005), Pujadas-Salvá (2010), and Liu et al. (2015). In turn, similar to *O. picridis*, an ellipsoid shape of the ovary was observed in *O. owerinii*, *O. minor*, *O. reticulate*, and *O. armena* (Zare et al. 2014; El Mokni et al. 2015; Sardar 2019). Different researchers argue that the nectary colour and ovary shape can be used as diagnostic traits in the genus *Orobanche* (Pujadas-Salvà and Arguimbau 2008; Pujadas-Salvá 2010; Frajman et al. 2013). In *O. picridis*, nectar was released through modified stomata, i.e. the so-called nectarostomata. This type of nectar secretion has been detected in many plant species from various botanical families (e.g. Papp et al. 2013; Antoń et al. 2017; Konarska 2017; Jachuła et al. 2018).

The nectaries in *O. picridis* were composed of epidermis and glandular parenchyma, whose cells contained large amyloplasts with numerous starch granules. It is known that, in obligate root parasites as *O. picridis*, assimilates taken up by modified roots, the so-called haustoria, from the host plant were transported via the vascular bundles of the parasite and accumulated as starch grains in ovary wall cells and next in glandular parenchyma cells. Since the *O. picridis* nectary was not equipped with vascular tissue, glucose molecules contained in the starch grains were transported to the nectary parenchyma cells probably via the symplastic route, as indicated by the presence of plasmodesmata in the cell walls of this tissue. During anthesis, there was undoubtedly a slow nocturnal breakdown of starch to simple sugars, which are the main components of prenectar. In turn, the electron-dense content in the intercellular spaces indicates an apoplastic route of secretion transport. Similarly, both types of nectar transport have been described in studies on the floral nectaries in representatives of other families, e.g. by Xiao (2000), Konarska and Weryszko-Chmielewska (2016), and Machado and Souza (2017). The large starch grains were present not only in glandular tissue cells but also in other tissues of the ovary wall as well as tissues of the inflorescence shoot and roots (unpublished data). It seems that the accumulation of such assimilates as starch grains taken up from the host plant by parasitic plants ensures constant availability and gradual utilisation thereof by ripening seeds and fruits and facilitates survival of underground parts (tubercles) in perennial *Orobanche*, e.g. *O. picridis*.

The *O. picridis* glandular parenchyma cells contained polymorphic plastids with dense osmiophilic material. Similar types of plastids were present in nectary cells in representatives of other families, e.g. Orchidaceae (De Melo et al. 2010; Kowalkowska et al. 2018) and Sapinadaceae (Weryszko-Chmielewska and Chwil 2017). The large amounts of smooth and rough ER profiles visible in the glandular parenchyma may have been involved in the synthesis of lipid and protein substances, respectively, and/or mediated the production and transport of nectar. These ER functions in nectary cells of other plants have been described by other researchers as well (Pacini and Nepi 2007; Paiva and Machado 2008; Paiva and Martins 2014).

The authors of the present study observed myelin-like multilamellar figures in glandular parenchyma vacuoles. Similar vacuolar inclusions were present in the nectary tissue cells in other plants (e.g. Antoń and Kamińska 2015; Possobom and Machado 2017). As reported by Wist and Davis (2005), this type of myelin-like figures may serve a lysosomal function and be involved in the continual degradation of senescing organelles during nectary secretion. Literature on holoparasitic plants does not provide any information on the structure and function of nectary glands.

### Trichome microstructure and histochemistry

The adaxial side of the sepals and petals as well as the pistil styles and ovaries in *O. picridis* flowers exhibited various densities of glandular trichomes; they were most abundant on the sepals and the least dense on the ovary and style. The multicellular capitate glandular trichomes had a head composed of a few to several dozen secretory cells arranged in a circle or built of two cell layers. In turn, there were sparse glandular trichomes on the stamens, whereas non-glandular trichomes were relatively abundant. Similar glandular trichomes were observed in several other *Orobanche* species by Sacchetti et al. (2003) and Hassan and El-Awadi (2009). Researchers investigating the location and density of trichomes in other *Orobanche* species showed the taxonomic importance of this trait (Table 1 and references wherein) which, similar to the different colour of trichomes (in particular their heads), can be used as a diagnostic criterion for identification of hardly recognisable and little differing taxa. For example, the glandular trichomes present on perianth elements were light yellow in *O. picridis*, violet in *O. owerini*, dark violet in *O. reticulata* (Zare and Dönmez 2013), yellow in *O. lammonensis* (Pujadas-Salvà and Arguimbau 2008), and white in *O. minor* and *O. purpurea* (Toma et al. 2007).

The presence of starch grains and the prevalence of mitochondria in the secretory cells of *O. picridis* glandular trichomes suggest high metabolic processes (transformation, secretion) occurring in these cells. It also indicates that starch is an energy source indispensable for these processes and/or can provide precursors of secretion components. This function of starch grains and mitochondria present in the secretory structures has also been reported by Radice and Galati (2003) and Machado et al. (2005). Various metabolites contained in the secretion of *O. picridis* trichomes were transported via the symplast from the stalk cells through the neck to the trichome head, which was confirmed by the presence of plasmodesmata between these cells and the results of histochemical assays and fluorescence studies. The secretory products penetrated through the walls in the distal part of the secreting cells, accumulated as oil droplets in the emerging subcuticular space, and were released after cuticle rupture. This type of secretion has also been observed in trichomes of representatives of other families, e.g. Asteraceae (Bombo et al. 2017), Lamiaceae (Haratym and Weryszko-Chmielewska 2017), Geraniaceae (Boukhris et al. 2013), Verbenaceae (Tozin et al. 2015), and Fabaceae (De Vargas et al. 2018).

The results of the histochemical assays and fluorescence studies revealed the presence of four groups of metabolites in the *O. picridis* glandular trichomes, i.e. polyphenols (tannins, flavonoids), lipids (acidic and neutral lipids, essential oil, sesquiterpenes, steroids), polysaccharides (neutral and acidic), and alkaloids (Table 3). The presence of a variety of bioactive compounds in the *O. picridis* trichomes suggests their multiple roles. Researchers agree that *Orobanche* spp. differ in the presence of metabolites in trichome cells: El-Akkad et al. (2002) and Hassan and El-Awadi (2009) detected the presence of lipid, lignin, phenolic, and suberin materials, Sacchetti et al. (2003) described the content of terpenes and flavonoids, Hegnauer (1990) identified iridoid glycosides and sesquiterpenes, Serafini et al. (1995) demonstrated the presence of phenylpropanoid glycosides, and Tóth et al. (2016) detected floral volatile organic compounds. The authors of the present study believe that the presence of alkaloids as well as tannins and flavonoids in the *O. picridis* trichomes provides protection to these plants against herbivore foraging and fungal or bacterial diseases. This is in agreement with the findings reported by other authors who regard alkaloids (Zúñiga and Corcuera 1986; Machado et al. 2005) and polyphenols (Ahmad et al. 2011; Santolamazza-Carbone et al. 2016) as the most effective repellents. In turn, other researchers have found that phenolic compounds contained in plants play a biological role, which is generally related to antifungal, antibacterial, and antifeedant activities (Bergau et al. 2015; Yamazaki and Lev-Yadun 2015). In turn, it has been reported that alkaloids detected in *O. egyptiaca* can be used in the treatment of hypertensive patients, especially in cases with heart failure complications (Sharaf and Youssef 1971). The presence of essential oil and sesquiterpenes in *O. picridis* glandular trichomes may increase the attractiveness of its flowers to insect visitors. As suggested by Harborne (1997b) and Machado et al. (2005), several biological activities are associated with essential oil, which have been recognised as phytoalexins, insect antifeedant, pheromones, defensive agents, allelochemicals, or signalling molecules. Furthermore, Li et al. (2018) have reported that glandular trichomes can be a barrier against atmospheric oxidative stress. In turn, Lahloub et al. (1991) have found that phenylpropanoid and iridoid glycosides contained in *O. ramosa* trichome secretion can be applied in phytotherapy and exhibit anti-inflammatory, antibacterial, antihypertensive, antitremor, and analgesic activities.

As reported by various authors, non-glandular trichomes constitute a mechanical barrier against adverse external factors such as UV-B radiation, extreme temperatures, and excessive water loss; yet, they also impede oviposition, foraging, and movement of herbivores (Werker 2000; Munien et al. 2015; Mitchell et al. 2016; Tozin et al. 2016). The authors of the present study postulate that the non-glandular trichomes present on *O. picridis* filaments may prevent nectar drying, as they protect nectar from the outer environment by close adherence to the ovary and corolla tube. On the other hand, the trichomes may be a convenient element for insect pollinators with short mouthparts and insufficient weight, which are unable to reach nectar accumulated deep in the corolla tube. Nectar moves along stamen trichomes up the corolla tube and becomes accessible to this group of insects as well. As shown by literature data, bumblebees, including short-tongued species, as well as wasps, Therevidae (Diptera), Colletidae, and Halictidae (Hymenoptera) are the main pollinators of *Orobanche* flowers (Jones 1991; Ollerton et al. 2007; Toth et al. 2013).

The presence or absence of glandular and non-glandular trichomes in *Orobanche* species as well as their distribution/location (Table 1), size, shape, structure, secretion pattern, and the composition of synthesised substances can be relevant taxonomic traits facilitating identification of species from this taxon. These traits are used in plant taxonomy to distinguish between closely related species, hybrids, or parasitic weeds (Adedeji et al. 2007; Salmaki et al. 2009; Morcelle et al. 2012; Redonda-Martínez et al. 2016).

To conclude, our studies indicate that *O. picridis* flowers exhibit a number of diagnostic traits that are relevant in studies of the evolution and relatedness between taxa of holoparasitic *Orobanche*. The micromorphological features of the calyx and corolla, pistil stigmata, nectaries, and trichome secretion differ between species, similar to the location and density of glandular and non-glandular trichomes present on the floral elements. Other taxonomically important traits, i.e. the structure of floral nectaries (mode of nectar production and secretion) and the ultrastructure of glandular trichomes, have been described in the genus *Orobanche* for the first time. The original investigations of the composition of the heterogeneous secretion of *O. picridis* trichomes describe another helpful trait for taxonomic studies of the genus *Orobanche*. It seems that further research elucidating the function of floral nectaries throughout the anthesis period and determining the quantity and composition of produced nectar is now required. Analyses of the composition of the *Orobanche* trichome secretion as a potential source of new active compounds with therapeutic properties seem promising as well.
